# Measurement and characterization of distinctive clinical phenotypes using the Frontotemporal Lobar Degeneration Module (FTLD-MOD)

**DOI:** 10.1002/alz.12098

**Published:** 2020-05-13

**Authors:** Tamar Gefen, Merilee A. Teylan, Lilah Besser, Emma Pollner, Anna Moshkovich, Sandra Weintraub

**Affiliations:** 1 Mesulam Center for Cognitive Neurology and Alzheimer’s Disease, Northwestern University Feinberg School of Medicine, Chicago, Illinois, USA; 2 Department of Psychiatry and Behavioral Sciences, Northwestern University Feinberg School of Medicine, Chicago, Illinois, USA; 3 National Alzheimer’s Coordinating Center, Department of Epidemiology, University of Washington, Seattle, Washington, USA; 4 Institute for Human Health and Disease Intervention (I-HEALTH), School of Urban and Regional Planning, Florida Atlantic University, Boca Raton, Florida, USA

**Keywords:** behavioral variant frontotemporal dementia, frontotemporal lobar degeneration, FTLD-MOD, primary progressive aphasia

## Abstract

**Introduction:**

The Frontotemporal Lobar Degeneration Module (FTLD-MOD) was designed as a research neuropsychological battery to evaluate clinical symptoms associated with FTLD. This study investigated whether the FTLD-MOD could differentiate between primary progressive aphasia (PPA) and behavioral variant frontotemporal dementia (bvFTD), two distinct FTLD-related syndromes.

**Methods:**

Retrospective analysis was conducted on data collected from the initial visit of 165 subjects with PPA, 268 with bvFTD, and 251 cognitively normal controls from the National Alzheimer’s Coordinating Center. Generalized linear models were used to compare group performance patterns on FTLD-MOD tasks of language, behavior, and memory.

**Results:**

PPA participants showed significantly poorer performances on all language tasks whereas bvFTD participants demonstrated poorer performances on most behavioral measures. There were no differences in memory performances. Descriptive data on participant groups are provided for reference.

**Discussion:**

Findings from this multi-center sample suggest that the FTLD-MOD can differentiate between distinctive clinical phenotypes commonly associated with FTLD.

## 1 | INTRODUCTION

Frontotemporal lobar degeneration (FTLD) constitutes the second most common cause of dementia under age 65.^1^ FTLD has been associated with a variety of distinctive clinical syndromes,^2^ including primary progressive aphasia (PPA), which is characterized by the early progressive loss of language with relative preservation of other cognitive modalities, including episodic memory.^3^ Behavioral variant frontotemporal dementia (bvFTD) constitutes another FTLD-related clinical dementia syndrome in which the most salient symptom is early, progressive decline in social comportment, judgment, and personality.^4^ As in PPA, memory is relatively spared in the initial stages of the bvFTD syndrome. Both PPA and bvFTD clinical syndromes are considered “atypical” in that they differ from the more typical amnestic dementia syndrome more commonly associated with Alzheimer’s disease (AD). In that syndrome, episodic memory loss is the most salient and earliest symptom.

The Alzheimer’s Disease Centers (ADC) program of the National Institute on Aging (NIA) initially designed the Uniform Data Set^5^ (UDS) to capture the key neuropsychological characteristics of Alzheimer’s dementia. A recent study found that the basic UDS neuropsychological test battery alone failed to differentiate between patients with *post mortem* AD versus FTLD neuropathology.^6^ The need to capture symptoms more commonly associated with FTLD prompted the NIA-ADC program to design a specialized module of the UDS,^5^ known as the FTLD module (FTLD-MOD). The FTLD-MOD consists of a series of psychometric assessments and surveys shown to be sensitive to the language and behavioral symptoms that are commonly associated with underlying *post mortem* FTLD neuropathology. The goal of the FTLD-MOD is to capture salient information about FTLD-related syndromes, like PPA and bvFTD, through curated measures not available in the AD-oriented UDS.

The clinical syndromes of PPA and bvFTD necessitate comprehensive evaluation to determine the relative salience of language and behavioral abnormalities. There are several clinical variants of PPA, each distinguished by predominant deficits in fluency, grammar, or semantics.^7^ The agrammatic variant of PPA is characterized by difficulties in morphology and syntax and is the subtype most often associated with the tau form of FTLD (tauopathy; FTLD-tau); the semantic variant (PPA-S) is most often associated with FTLD neuropathology characterized by abnormalities in the tar DNA binding protein (FTLD-TDP); and the logopenic variant is most commonly associated with AD.^8^ The language tests included as part of the FTLD-MOD were therefore designed to measure different aspects of grammar, word knowledge, and word-finding difficulty affected in patients with PPA. In bvFTD, progressive changes in empathy, self-monitoring, and personality/character have only been studied experimentally with few uniform and standardized instruments to directly assess these changes. The Frontal Behavior Inventory^9^ is one informant-rated questionnaire that evaluates the type and extent of behavioral changes that has been commonly used in research on bvFTD. Another questionnaire, the Frontal Systems Behavioral Scale (FrSBe), is a standardized instrument to assess patient and informant ratings of past and current behavior to determine changes from baseline functioning.^10^ These instruments have been useful but lack the targeted focus on key diagnostic symptoms associated with research clinical consensus criteria for the diagnosis of bvFTD.^11^ Thus, the FTLD-MOD assembled research instruments shown to be sensitive to the highly nuanced behavioral, social, and interpersonal changes experienced by patients with bvFTD.

Since the addition of the FTLD-MOD, there has been some evidence showing its promise in distinguishing clinically diagnosed syndromes of amnestic dementia of the Alzheimer type versus bvFTD.^12^ The current study aimed to determine whether specific FTLD-MOD measures targeting language functions and social behaviors could differentiate between individuals with clinically diagnosed PPA and those with

## 2 | METHOD

This is a retrospective analysis of data obtained from a sample in the National Alzheimer Coordinating Center (NACC) database. The sample included participants assessed between September 2005 and June 2017 at the NIA-funded ADCs. The analytic sample was restricted to participants’ first UDS visit where they completed the supplemental FTLD-MOD and met one of the following diagnostic criteria: (a) clinically diagnosed as cognitively normal and a global Clinical Dementia Rating (CDR) scale^13^ score of 0 or (b) primary clinical diagnosis of dementia and bvFTD or PPA, dementia syndromes with high likelihood of one or another form of FTLD as the etiology. The diagnosis of an FTLD-related syndrome had been made without considering performance on the FTLD-MOD and was based on up-to-date research diagnostic criteria for bvFTD^11^ and for PPA^3,8^ and according to procedures of the UDS (https://www.alz.washington.edu). FTLD-MOD performance data were available from 165 participants with a primary clinical diagnosis of PPA, 268 participants with a clinical diagnosis of bvFTD, and 251 cognitively normal controls.

### 2.1 | The FTLD-MOD of the UDS

The FTLD-MOD neuropsychological battery is a collection of commonly used clinical measures, in addition to some developed for the purpose of targeted research studies on FTLD-related disorders. Language tests include two letter fluency tasks (F and L); a test of single word reading of regular and irregular words; a test of sentence reading and repetition; a test of noun and verb naming from the Northwestern Naming Battery;^14^ a test of non-oral grammatical sentence construction (Northwestern Anagram Test^15^); a test of single word comprehension; and a test of nonverbal semantic associates from the Northwestern Naming Battery.^14^ Five questionnaires measure social/behavioral symptoms. Three are completed by an informant rating observed aspects of behavior manifested by the patient: (1) the Behavior Inhibition Scale^16^ evaluates the patient’s inhibitory and excitatory tendencies; (2) the Interpersonal Reactivity Index (created by Mark Davies, PhD for the NACC FTLD-MOD) questions the informant about empathic concern and perspective-taking in everyday social interactions; and (3) the Revised Self-Monitoring Scale^17^ measures sensitivity to the expressive behavior of others and the ability to monitor self-presentation. Typically, patients with bvFTD do not have adequate insight to appreciate deficits in social and interpersonal faculties;^18^ given this, collateral information obtained by an informant (commonly, a family member or caregiver) and by clinical observation are necessary to evaluate the extent and type of behavioral impairment. The Social Norms Questionnaire (created by Katherine Rankin, PhD, for the NACC FTLD-MOD) is administered to the patient and assesses the degree to which the subject understands and identifies widely accepted social boundaries. The Social Behavior Observer Checklist (created by Katherine Rankin, PhD, for the NACC FTLD-MOD) notes the frequencies of observed spontaneous behaviors during the clinical evaluation from the perspective of the examiner, including odd or inappropriate behavior. Table 1 provides descriptions of the FTLD-MOD with examples of items taken from each behavioral measure. See https://www.alz.washington.edu for FTLD-MOD documentation.

### 2.2 | Statistical analysis

Multivariable linear regression models with Bonferroni corrections were used to compare performance patterns between PPA, bvFTD, and control groups on memory, language, and behavioral measures administered as part of the FTLD-MOD. Models were adjusted for age, education, and sex. Generalized estimating equations accounted for clustering of the data by ADC. Statistical significance was determined with an *α* level of 0.05.

## 3 | RESULTS

Table 2 shows the demographics of the three groups that were included in the analysis, along with CDR and Mini-Mental State Examination (MMSE)^19^ performance at initial administration of the FTLD-MOD. As expected, PPA (mean [M] = 18.2; standard deviation [SD] = 8.8) patients scored significantly lower than the bvFTD group (M = 22.3; SD = 6.9) on the MMSE. Age at initial symptom onset was significantly younger in individuals with bvFTD (M = 57.8; SD = 8.5) compared to those with PPA (M = 61.7; SD = 8.2; *P* < 0.05); there were no age differences between PPA or bvFTD groups compared to the control sample (M = 57.2; SD = 14.9). There were no differences in level of education among participants with PPA (M = 15.7; SD = 2.9), bvFTD (M = 15.6; SD = 3.2), and cognitively normal controls (M = 15.6; SD = 2.6). The proportion of males to females in the bvFTD group was significantly larger (61.9% male) compared to the PPA group (51.5% male) and cognitively normal control group (47.0% male; *P* < 0.05). The reason for sex differences in the bvFTD group is unknown and in contrast to larger population-based prevalence studies that find no apparent predisposition for frontotemporal dementias based on sex.^20^

Controls had higher scores (ie, showed fewer errors or endorsed fewer symptoms) on nearly all language and behavioral subtests of the FTLD-MOD compared to PPA and bvFTD groups. The exceptions were scores on the Word-Reading: Regular Words task and, more surprisingly, the Behavior Inhibition Scale (Observer). Control group performance on the memory subtests of the FTLD-MOD was higher compared to both clinical groups (*P* < 0.05), and PPA patients did not significantly differ from bvFTD patients on either immediate or delayed memory subtest scores (see Tables 3 and 4).

**FIGURE 1:**
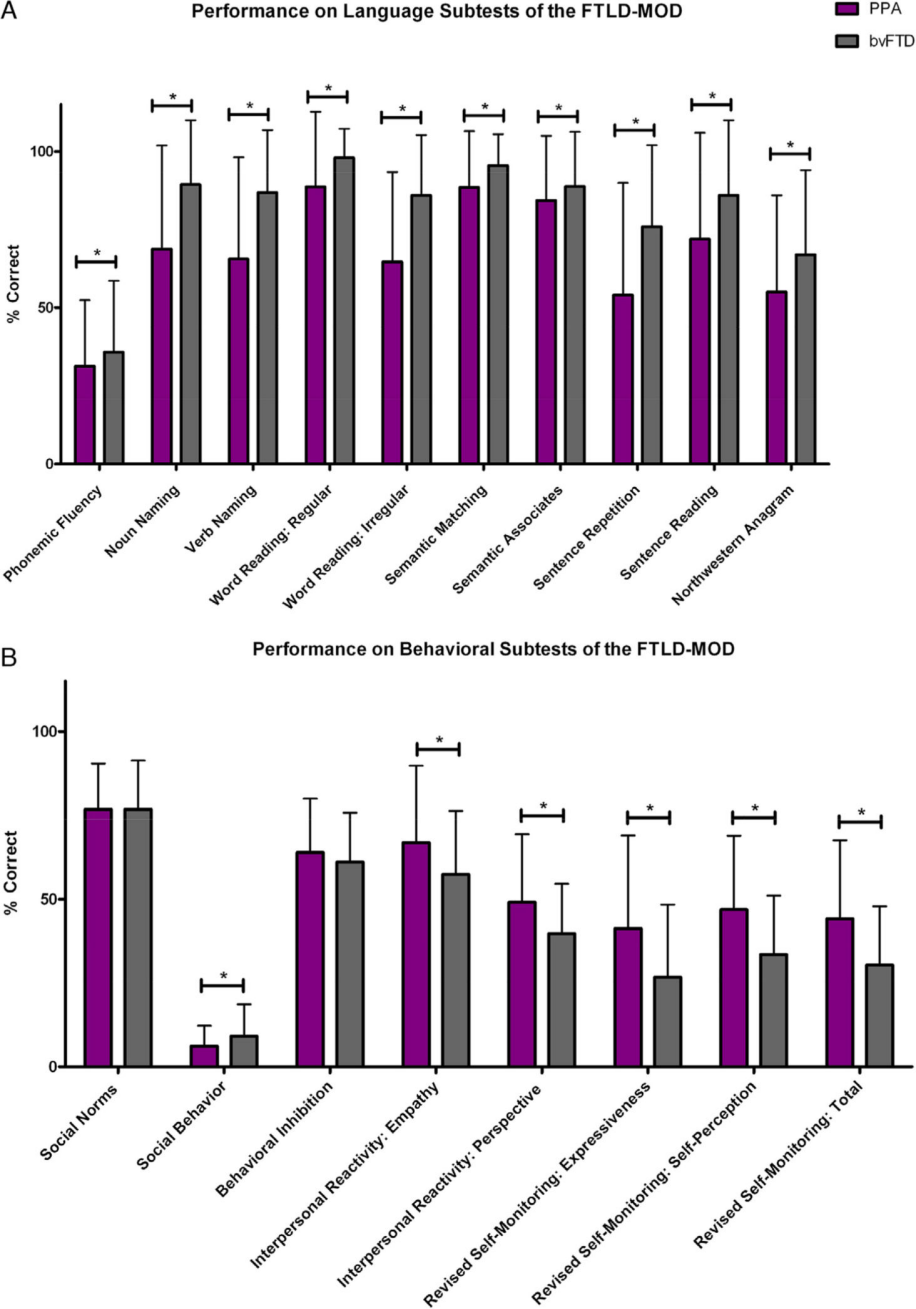
A, Mean performance (total score) on language-related measures of the FTLD-MOD by clinical diagnosis. * = statistically significant difference between PPA and bvFTD at *P* < 0.05; error bars = standard deviation. B, Mean performance (indicated as percent “correct,” which signifies a higher tendency toward intact or socially acceptable behavioral performances) on behavior-related measures of the FTLD-MOD by clinical diagnosis. * = statistically significant difference between PPA and bvFTD at *P* < 0.05; error bars = standard deviation.

PPA patients performed significantly worse than bvFTD patients on all language subtests of the FTLD-MOD including measures of fluency (Phonemic Fluency: Total F/L), naming (noun and verb naming), reading (regular and irregular word reading), word-knowledge and single word object meaning (Semantic Word-Picture Matching Test and Semantic Associates Test), sentence repetition and reading, and grammar (Northwestern Anagram Test; *P* < 0.05). See Figure 1A; scores were transformed for the purpose of depiction to reflect the percentage of items scored as correct (percent correct).

Those with bvFTD demonstrated more symptoms compared to those with PPA on subtests that measure social norms and behavior (Social Behavior Observer Checklist), interpersonal reactivity and sensitivity (Interpersonal Reactivity Index), and self-perception and monitoring (Revised Self-monitoring Scale Total Score; *P* < 0.05, per test). However, those with bvFTD (M = 16.9, SD = 3.2) did not show significantly different scores on the Social Norms Questionnaire Total Score compared to those with PPA (M = 16.9, SD = 3.0). Again, as mentioned, controls had fewer symptoms on all behavioral subtests of the FTLD-MOD compared to both clinical groups (*P* < 0.05), with the exception of the Behavior Inhibition Scale (Observer)–Total Score; interestingly, patients with PPA (M = 17.9, SD = 4.5) showed significantly higher scores–signifying a slightly greater tendency toward inhibitive or withdrawal behaviors–on this observer scale compared to controls (M = 16.7, SD = 4.0). There were no differences between the bvFTD group (M = 17.1, SD = 4.1) and other groups on this scale. See Figure 1B; scores are also reflective of percent correct. Mean performances and adjusted mean differences in FTLD-MOD test scores from this robust sample are presented in Tables 3 and 4.

## 4 | DISCUSSION

Frontotemporal lobar degeneration constitutes a heterogeneous class of pathologic species, each of which can lead to a variety of dementia syndromes.^21^ Clinicians and scientists are thus confronted with a major challenge when attempting to diagnose FTLD in a living patient due to the wide range of associated clinical symptoms and pathologies. In comparison to patients with AD, those with FTLD are relatively underserved as a result of this complex clinicopathologic heterogeneity as it leads to uncertainty surrounding diagnosis and there are no currently available disease biomarkers.

PPA became one of the first syndromes to show that the same clinical phenotype can be caused by heterogeneous pathologies.^22,23^ Three major neuropathologic entities account for the majority of PPA cases: AD, frontotemporal lobar degeneration with TDP-43 inclusions (FTLD-TDP), and frontotemporal lobar degeneration with tau inclusions (FTLD-tau).^21^ Like PPA, bvFTD is also a clinically heterogeneous syndrome, but the majority of cases show either FTLD-tau or FTLDTDP. AD neuropathology can be associated with bvFTD but this is much less common.^24^ These clinicopathologic relationships, however, are probabilistic rather than absolute.^25^ Given probabilistic clinicopathologic relationships, and a lack of *ante mortem* biomarkers, it has become increasingly critical to identify and highlight the efficacy of standardized clinical tools in the early diagnosis of FTLD-related syndromes like PPA and bvFTD. The FTLD-Module (FTLD-MOD) of the UDS was designed as a research instrument to measure the language impairments and behavioral changes experienced by patients with FTLD-related diseases. The current study specifically investigated whether specific measures targeting language and social behaviors included as part of the FTLD-MOD can adequately differentiate between the clinical symptoms associated with bvFTD versus PPA.

In this study, analysis of FTLD-MOD performances between large groups of participants with PPA, bvFTD, and cognitively normal controls yielded three main findings. First, as anticipated, cognitively normal controls performed significantly better on nearly all language tests of the FTLD-MOD and demonstrated fewer behavioral symptoms compared to PPA and bvFTD groups. Second, bvFTD patients demonstrated greater behavioral symptoms than PPA patients on nearly all behavioral subtests, while PPA patients performed significantly worse than bvFTD patients on all language subtests, without exception. Finally, and in accordance with initial stages of symptom presentation in PPA and bvFTD, both patient groups did not differ from one another on memory measures. In general, with the exception of performance patterns on the Behavioral Inhibition Scale and the Social Norms Questionnaire, language-based assessments and behavioral surveys that comprise the FTLD-MOD appear to differentiate between distinctive clinical phenotypes most commonly associated with FTLD.

There are two possibilities as to why the Behavioral Inhibition Scale showed significant differences between the PPA and bvFTD group, such that–perhaps unexpectedly–the PPA group demonstrated a greater number of symptoms. One is that the Behavioral Inhibition Scale is a 7-item questionnaire based on informant reports, which are susceptible to variability due to subjective responding.^26,27^ The second, and more plausible possibility, is that PPA participants are indeed more likely to show a tendency toward behavioral inhibition as measured by the Behavioral Inhibition Scale; that is, specific features that are consistent with traits such as social withdrawal, anxiety, and introversion. One recent study showed that the inability to communicate in PPA was related to high likelihood of depression, anxiety, irritability, and apathy, among other neuropsychiatric symptoms.^28^ PPA patients, compared to those with bvFTD, also showed similar scores on the Social Norms Questionnaire, which assesses the degree to which the research participant understands widely accepted social norms. The scale is based on self-report and presented in a yes/no question format, which may pose problems for PPA patients, particularly those with difficulties in comprehension and grammar.^29^ These hypotheses can be tested carefully in the future by assessing the specificity and sensitivity of the Behavioral Inhibition Scale and the Social Norms Questionnaire measures against other valid psychometric measures.

Findings from this study will be helpful in supporting diagnostic specificity and in clarifying clinicopathologic relationships with granularity and nuance. A central challenge in the field of neurodegenerative disorders concerns the correspondence between phenotypic features of dementia and molecular pathology,^30^ and the FTLD-MOD appears well suited to detect the subtle differences in PPA versus bvFTD syndromes. Future studies will focus on investigating the utility of the FTLD-MOD to predict underlying pathologic substrates. As clinicopathologic relationships become more confidently established, disease-specific diagnostic tools and treatments can develop to further serve the FTLD community.

## Figures and Tables

**TABLE 1:** FTLD-MOD: domains of measurement and brief descriptions of assessment

FTLD-MOD battery subtests	Domains of measurement/descriptions of assessment
***Memory***	
Benson Complex Figure Copy—Immediate Benson Complex Figure Copy—Delayed	Visuoconstructional and visual memory functions (encoding and recall)
***Language***	
Phonemic Fluency	Spontaneous word generation to letters F and L
Noun and Verb Naming	Naming of single objects and actions
Word reading: Regular and Irregular Words	Reading regular and irregularly spelled words aloud
Semantic Word-Picture Matching Test	Auditory word recognition and frequency of semantic errors in word comprehension
Semantic Associates Test	Knowledge of the meaning of pictured objects
Sentence Repetition Test	Oral repetition of sentence-length utterances
Sentence Reading Test	Full sentence oral reading
Northwestern Anagram test	Grammatical sentence construction
***Behavioral/Social***	
Social Behavior Observer Checklist	Evaluates observed frequencies of spontaneous behaviors during the clinical evaluation, including odd or inappropriate behaviors Example: *“Was [the subject] overly disclosing or inappropriately familiar?”* (Not at all/A little bit/Moderately/Severely)
Interpersonal Reactivity Index	Measures empathic concern and perspective-taking in everyday social interactions Example: *“The subject is likely to try to understand others better by imagining how things look from their perspective.” [Scale 1 (Does NOT describe well) to 5 (Describes VERY well)].*
Revised Self-Monitoring Scale	Measures sensitivity to the expressive behavior of others and the ability to monitor self-presentation Example: *“The subject is often able to correctly read people’s true emotions through their eyes.”* [Scale 0 (Certainly, always false) to 5 (Certainly, always true)].

Note: These summarized descriptions are based on the freely available individual FTLD-MOD forms and documentation (https://www.alz.washington.edu).

Abbreviation: FTLD-MOD, Frontotemporal Lobar Degeneration Module

**TABLE 2:** Participant demographic and clinical screening measures (mean; SD) among participants with PPA, bvFTD, and normal controls

	PPA	bvFTD	Cognitively normal controls
N	165	268	251
Age (years)	66.8 (8.1)^a^	63.9 (8.2)^a^	57.2 (14.9)
% Male	51.5%^a^	61.9%^a,b^	47.0%^b^
Education (years)	15.7 (2.9)	15.6 (3.2)	15.6 (2.6)
Age of onset (years)	61.7 (8.2)^a^	57.8 (8.5)^a^	N/A
MMSE	18.2 (8.8)^a,b^	22.3 (6.9)^a,b^	29.0 (1.4)^b^
Global CDR (N, %)			
0	15 (9.1)	2 (0.8)	251 (100.0)
0.5	65 (39.4)	69 (25.8)	0 (0.0)
1	50 (30.3)	120 (44.8)	0 (0.0)
2	24 (14.6)	58 (21.6)	0 (0.0)
3	11 (6.7)	19 (7.1)	0 (0.0)

a Significant difference between PPA and bvFTD at *p*<0.05.

b Significant difference at *P* < 0.05 compared to normal controls.

Abbreviations: bvFTD, behavioral variant frontotemporal dementia; CDR, Clinical Dementia Rating scale; FTLD-MOD, Frontotemporal Lobar Degeneration Module; MMSE, Mini Mental State Examination; PPA, primary progressive aphasia; SD, standard deviation

**TABLE 3:** FTLD-MOD Subtest Raw Scores (Mean; SD) among participants with PPA, bvFTD, and normal controls

FTLD-MOD battery subtest (max score)	PPA	bvFTD	Cognitively normal controls
***Memory***			
Benson Complex Figure Immediate (17)	13.6 (4.1)	13.6 (3.9)	15.8 (1.7)
Benson Complex Figure Delay (17)	7.1 (4.5)	7.2 (4.8)	12.5 (2.9)
***Language***			
Phonemic fluency: Total F & L (38^a^ )	11.9 (8.0)	13.6 (8.7)	28.9 (8.7)
Noun Naming (16)	11.0 (5.3)	14.3 (3.3)	15.9 (0.4)
Verb Naming(16)	10.5 (5.2)	13.9 (3.2)	15.9 (0.4)
Word Reading: Regular Words (15)	13.3 (3.6)	14.7 (1.4)	15.0 (0.1)
Word Reading: Irregular Words (15)	9.7 (4.3)	12.9 (2.9)	14.5 (1.0)
Semantic Word-Picture Matching Test (20)	17.7 (3.6)	19.1 (2.0)	19.9 (1.3)
Semantic Associates Test (16)	13.5 (3.3)	14.2 (2.8)	16.0 (0.2)
Sentence Repetition Test (5)	2.7 (1.8)	3.8 (1.3)	4.5 (0.6)
Sentence Reading Test (5)	3.6 (1.7)	4.3 (1.2)	4.9 (0.4)
Northwestern Anagram Test (10)	5.5 (3.1)	6.7 (2.7)	9.2 (1.3)
***Behavioral/Social***			
Social Norms Questionnaire Total Score (22)	16.9 (3.0)	16.9 (3.2)	20.0 (1.5)
Social Behavior Observer Checklist Total Score (105)	6.5 (6.3)	9.5 (10.0)	1.6 (2.7)
Behavior Inhibition Scale (Observer) Total score (28)	17.9 (4.5)	17.1 (4.1)	16.7 (4.0)
Interpersonal Reactivity Index			
Empathic Concern Score (35)	23.4 (8.0)	20.1 (6.6)	28.3 (5.1)
Perspective Taking Score (35)	17.2 (7.1)	13.9 (5.2)	24.5 (6.2)
Revised Self-Monitoring Scale			
Sensitivity to Social Emotional Expressiveness Score (30)	12.4 (8.3)	8.0 (6.5)	21.3 (5.3)
Ability to Modify Self-Perception Score (35)	16.4 (7.7)	11.7 (6.2)	24.4 (5.6)
Total score (65)	28.7 (15.2)	19.7 (11.4)	45.7 (10.0)

Numbers in parentheses next to the subtest indicate maximum score.

a This value was used as maximum value (1SD above mean for controls).

Abbreviations: bvFTD, behavioral variant frontotemporal dementia; FTLD-MOD, Frontotemporal Lobar Degeneration Module; PPA, primary progressive aphasia

**TABLE 4:** Adjusted mean differences in FTLD-MOD test scores comparing diagnostic groups

	PPA versus bvFTD (ref)		PPA versus controls (ref)		bvFTD versus controls (ref)	
FTLD-MOD battery subtest	*β* est	Std Err	*β* est	Std Err	*β* est	Std Err
***Memory***						
Benson Complex Figure Immediate	0.11	0.38	**−2.16**	**0.36**	**−2.27**	**0.29**
Benson Complex Figure Delay	0.18	0.44	**−5.15**	**0.54**	**−5.33**	**0.37**
***Language***						
Phonemic fluency:Total F&L	**−1.96**	**0.77**	**−17.43**	**1.41**	**−15.50**	**1.58**
Noun Naming	**−3.31**	**0.52**	**−4.91**	**0.36**	**−1.60**	**0.19**
Verb Naming	**−3.45**	**0.45**	**−5.43**	**0.38**	**−1.98**	**0.18**
Word Reading: Regular Words	**−1.48**	**0.48**	**−1.75**	**0.56**	−0.27	0.14
Word Reading: Irregular Words	**−3.36**	**0.37**	**−4.93**	**0.40**	**−1.56**	**0.18**
Semantic Word-Picture Matching Test	**−1.47**	**0.25**	**−2.19**	**0.22**	**−0.73**	**0.09**
Semantic Associates Test	**−0.68**	**0.23**	**−2.51**	**0.14**	**−1.83**	**0.12**
Sentence Repetition Test	**−1.18**	**0.19**	**−1.89**	**0.18**	**−0.71**	**0.08**
Sentence Reading Test	**−0.70**	**0.10**	**−1.31**	**0.09**	**−0.61**	**0.05**
Northwestern Anagram Test	**−1.31**	**0.49**	**−3.79**	**0.39**	**−2.48**	**0.16**
***Behavioral/Social***						
Social Norms Questionnaire Total Score	−0.18	0.47	**−3.22**	**0.29**	**−3.04**	**0.30**
Social Behavior Observer Checklist Total Score	**−2.82**	**1.06**	**5.39**	**0.60**	**8.22**	**1.05**
Behavior Inhibition Scale (Observer) Total Score	0.80	0.46	**1.48**	**0.50**	0.68	0.30
Interpersonal Reactivity Index						
Empathic Concern Score (35)	**3.16**	**0.53**	**−4.70**	**0.62**	**−7.85**	**0.54**
Perspective Taking Score (35)	**3.30**	**0.44**	**−7.39**	**0.56**	**−10.69**	**0.49**
Revised Self-Monitoring Scale						
Sensitivity to Social Emotional Expressiveness Score (30)	**4.46**	**0.59**	**−8.79**	**0.66**	**−13.20**	**0.58**
Ability to Modify Self-Perception Score (35)	**4.91**	**0.67**	**−8.17**	**0.92**	**−13.08**	**0.77**
Total Score (65)	**9.41**	**1.18**	**−17.13**	**1.49**	**−26.55**	**1.19**

Notes: Models adjusted for age, sex, and education. **Bolded** cells show statistically significant mean differences with Bonferroni correction at *P* < 0.05. The second group in the column header is the reference group (“ref”) in the comparison and negative beta estimates indicate that the comparison group scored significantly lower than the reference group.

Abbreviations: bvFTD, behavioral variant frontotemporal dementia; FTLD-MOD, Frontotemporal Lobar Degeneration Module; PPA, primary progressive aphasia

## RESEARCH IN CONTEXT

### Systematic review

The authors reviewed the current literature published on the use and utility of Frontotemporal Lobar Degeneration Module (FTLD-MOD) of the National Alzheimer’s Coordinating Center. There were a small number of studies that investigated the use of the FTLD-MOD to characterize atypical dementia phenotypes, and none existed that specifically examined whether its measures could differentiate between PPA versus bvFTD.

### Interpretation

Findings suggest that the FTLD-MOD can differentiate between distinctive clinical phenotypes commonly associated with FTLD. These data can be useful to the Alzheimer’s Disease Centers program of the National Institute on Aging (NIA), which exist to characterize and investigate Alzheimer’s disease and related neurodegenerative disorders like FTLD.

### Future directions

Future studies will focus on examining the utility of the FTLD-MOD to predict underlying pathologic substrates. As clinicopathologic relationships become more confidently established, disease-specific diagnostic tools and treatments can develop to further serve the FTLD community.
